# Association between the use of Accredited Social Health Activist (ASHA) services and uptake of institutional deliveries in India

**DOI:** 10.1371/journal.pgph.0002651

**Published:** 2024-01-16

**Authors:** Sujata Mishra, Susan Horton, Zulfiqar A. Bhutta, Beverley M. Essue

**Affiliations:** 1 Institute of Health Policy, Management and Evaluation, University of Toronto, Toronto, Canada; 2 School of Public Health Sciences, University of Waterloo, Waterloo, Canada; 3 Centre for Global Child Health, Hospital for Sick Children, Toronto, Canada; 4 Institute for Global Health & Development, The Aga Khan University, Karachi, Pakistan; Indian Institute of Technology Bombay, INDIA

## Abstract

This study examines the impact of accredited social health activists (ASHAs), on increasing rates of institution-based deliveries among Indian women with a specific focus on the nine low-performing, empowered action group states and Assam (EAGA) in India. Using the latest round of the National Family Health Survey-V (2019–21), we first investigate the association between the use of ASHA services and socio-demographic attributes of women using a multivariate logistic regression. We then use propensity-score matching (PSM) to address observable selection bias in the data and assess the impact of ASHA services on the likelihood of institution-based deliveries using a generalized estimating equations model. Of the 232,920 women in our sample, 55.5% lived in EAGA states. Overall, 63.3% of women (70.6% in EAGA states) reported utilizing ASHA services, and 88.6% had an institution-based delivery (84.0% in EAGA states). Younger women from the poorest wealth index were more likely to use ASHA services and women in rural areas had a two-fold likelihood. Conversely, women with health insurance were less likely to use ASHA services compared to those without. Using PSM, the average treatment effect of using ASHA services on institution-based deliveries was 5.1% for all India (EAGA = 7.4%). The generalized estimating equations model indicated that the use of ASHA services significantly increased the likelihood of institution-based delivery by 1.6 times (95%CI = 1.5–1.7) for all India (EAGA = 1.8; 95%CI = 1.7–1.9). Our study finds that ASHAs are effective in enhancing the uptake of maternal services particularly institution-based deliveries. These findings underscore the necessity for continual, systematic investments to strengthen the ASHA program and to optimize the program’s effectiveness in varied settings that rely on the community health worker model, thereby advancing child and maternal health outcomes.

## Introduction

Despite India’s remarkable economic progress, the maternal and child mortality burden remains high, reflecting persistent challenges in ensuring equitable access to essential primary healthcare (PH) services [1,2]. Socioeconomic disparities further compound this issue, particularly in the utilization of crucial services such as ante-natal care (ANC) and institution-based deliveries [2,3]. The uneven distribution of the maternal and child mortality burden is evident across geographical regions and socio-economic strata, highlighting the need for targeted interventions. Notably, certain states in India and more specifically the nine low-performing states collectively called the Empowered Action Group and Assam (EAGA) (including Uttar Pradesh, Bihar and Jharkhand) have lower uptake of institution-based deliveries and a disproportionately larger number of maternal and child deaths. This differs from the economically advanced states of Kerala and Tamil Nadu which have higher access to and utilization of PH [2,4]. Furthermore, the vulnerability of low socioeconomic status groups exacerbates the mortality burden, as these populations often underutilize essential maternal care services [5]. Home-based deliveries, in the absence of skilled birth attendants, particularly in rural areas and among women with low socio-economic status, are not uncommon leading to a sizable proportion of preventable maternal and infant deaths [2,6].

Global evidence highlights the transformative potential of community health workers (CHWs), who act as drivers of improved access to and coverage of basic health services [7,8]. In India like in most low- and middle-income countries (LMICs), CHWs target underserved populations, and educate and facilitate the uptake of PH services like ANC and institution-based deliveries, thereby reducing health inequities in access to essential PH [8–10].

In the last two decades, India has invested significantly in safe motherhood interventions, notably through the Janani Suraksha Yojana (JSY) which provides conditional cash transfers to promote institution-based deliveries and enhance PH access for women in their pre-and post-natal period [11]. Another key intervention was the introduction of accredited social health activists (ASHAs) in 2005 under the National Health Mission (NHM). Recruited locally, ASHAs are culturally sensitive trained volunteers and educators whose aim is to improve maternal and child outcomes [12]. Part of the work of ASHAs includes identifying pregnant women within their jurisdiction and facilitating registration for ANC which provides a crucial link between the primary healthcare centre and expectant mothers. They actively implement the JSY scheme by counselling women on institution-based delivery, assisting in the selection of suitable health facilities for delivery, escorting them to predetermined centers, and facilitating the JSY cash transfer disbursement post-delivery [11,12]. Furthermore, ASHAs provide breastfeeding counselling, arrange postnatal visits within the first week of delivery, and monitor the mother and child’s health [12–14]. The ASHA program is recognized as one of the largest publicly funded CHW initiatives globally, with a workforce exceeding one million, deployed across various states in India [15].

Since its inception, only a limited number of studies have explored the likelihood of engaging with ASHA services and its influence on the utilization of maternal health services, specifically institution-based deliveries. Additionally, significant variations in the receipt of ASHA services exist, with factors such as age, education, caste, and religion playing a role in influencing the likelihood of using these services [15,16]. Evidence for the effectiveness and impact of ASHAs in improving healthcare utilization, specifically the uptake of institution-based delivery services, is inconclusive [15,17,18]. A systematic review by Scott and colleagues (2019) summarized ten years of published research on the ASHA program and found mixed evidence on the role of ASHAs in routine programmes, including improving healthcare utilization [17].

Some studies show a positive impact of ASHA services, including enhanced health equity (defined as improved access and utilization of essential healthcare services for all), increased likelihood of institution-based deliveries [15,19,20], and reduced perinatal mortality rates [10]. However, contrasting findings exist in other studies. For instance, Wagner et.al. (2018) found that increased ASHA placement within districts did not lead to any significant change in institution-based deliveries [21]. In a four-state study, Koehn et.al. (2020) concluded that mothers who received ASHA visits were significantly less likely to have an institutional delivery [22]. Moreover, the perceived role of ASHA workers among village community members was often limited, with ASHAs being viewed more as facilitators than community health workers [18]. Previous studies have predominantly been conducted in limited geographic regions, often confined to a few districts or specific states [18,20,22]. Additionally, they have been constrained in their consideration of selection bias, whereby women who utilize ASHA services and opt for institution-based deliveries may differ in socioeconomic attributes from those who do not use these services [15,19].

**Fig 1:**
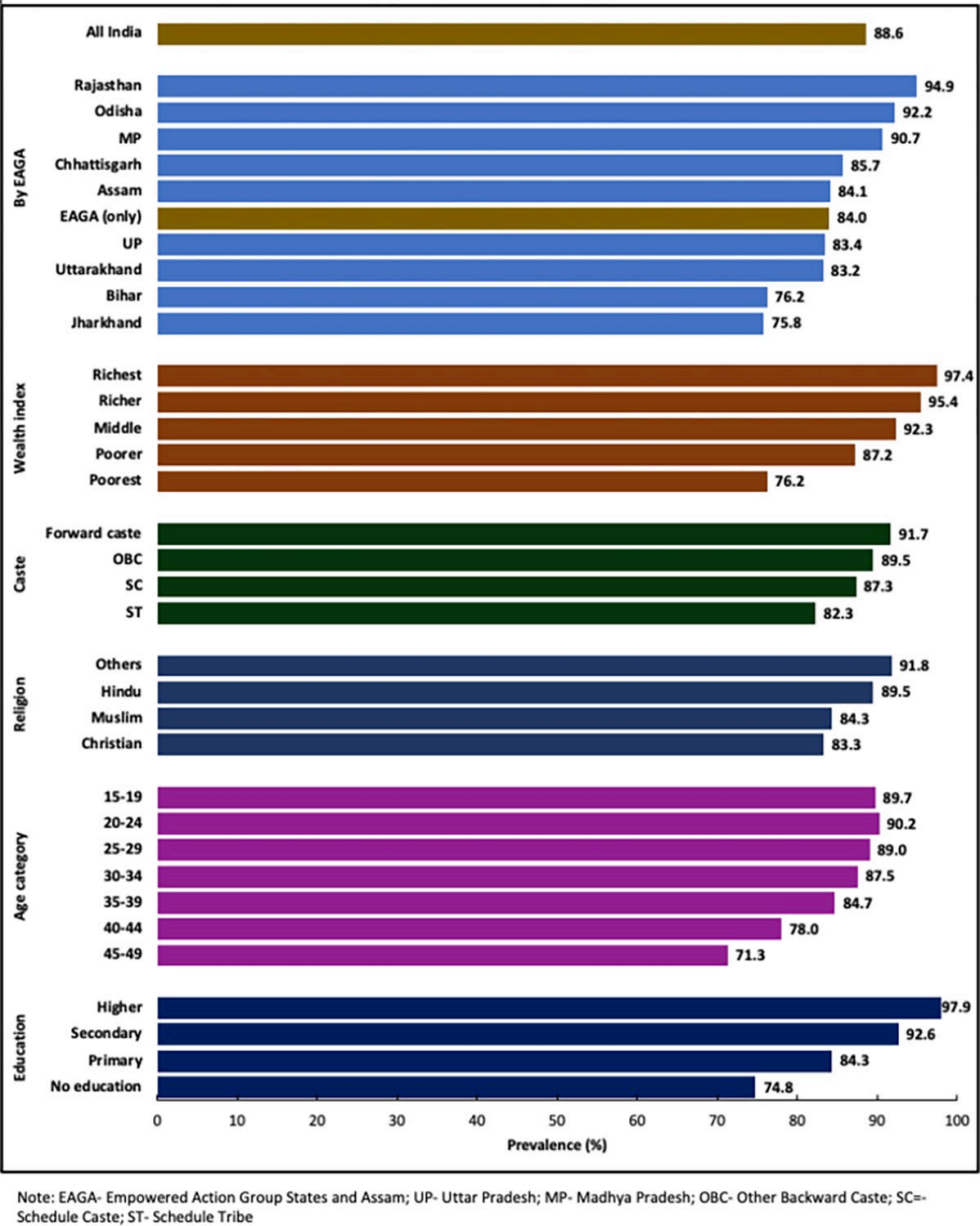
Weighted prevalence of institution-based deliveries by mother’s characteristics in India (NFHS V-2019-2021)).

The variance in these findings suggests there is a need for more conclusive and reliable evidence to establish the association between ASHA services and institution-based deliveries in the Indian context, using more recent rounds of national survey data. Additionally, it is crucial to address the issue of selection bias in the study design. Studies of this nature hold particular significance for low-performing EAGA states and for low socioeconomic status groups, where disparities in institution-based deliveries are more pronounced (Fig 1).

Furthermore, considering the scope of and investment into the ASHA program, it is essential to gather clear evidence of its impact not only for the Indian government but also for other LMICs deploying similar CHW programs. This will lay the groundwork for ongoing monitoring of the program’s impact. Our study aims to provide valuable insights into the effectiveness of ASHA programs in improving institution-based deliveries and contribute to evidence-based policy recommendations and intervention development to enhance maternal and child healthcare in India and potentially other LMICs.

This study investigates whether the ASHA program increases rates of institution-based deliveries and advances health equity among Indian women with a focus on the high-burden, low-performing EAGA states. The study objectives are two-fold: Objective 1: Assess the relationships between the use of ASHA services and the individual characteristics of women. Objective 2: Investigate the association between institution-based deliveries and the use of ASHA services, accounting for underlying socio-demographic attributes.

## Method and empirical analyses

### Ethics statement

Ethical approval was received from the Health Sciences Research Ethics Board, University of Toronto (Approval No.- RIS HPN 44296).

The reporting in this study is guided by the Strengthening and Reporting of Observational Studies in Epidemiology (STROBE Statement) [23]. See S1 Table for details.

### Data source

This analysis uses the latest round of the National Family Health Survey-V (NFHS; 2019–21) in India [24]. The sample design ensures representativeness at the national and state levels, enabling stratified analysis by states and geographic regions. The NFHS-V survey collects primary data using four types of questionnaires (i.e., the household, women, men, and biomarker). The present study specifically uses data captured by the women’s questionnaire which provides information about ever-married women between the ages of 15–49 years. This questionnaire includes the respondent’s birthing history, reproductive health, and antenatal, postnatal and delivery care. For women with multiple births in the survey, we restrict the study sample to the last birth in the 5 years preceding the survey date as the data are more complete for this population. National (referred to as ‘all India’ here-on) estimates are presented alongside estimates from the nine EAGA states that are grouped in all analyses.

The EAGA states are Bihar, Chhattisgarh, Jharkhand, Madhya Pradesh, Orissa, Rajasthan, Uttaranchal, Uttar Pradesh, and Assam. These states lag in the demographic transition and have the highest maternal and infant mortality rates in the country [1,2]. This group was constituted by NHM for the preparation and implementation of area-specific health interventions and makes up 45% of the total population of India and 61% of those living below the poverty line [25].

### Variables and measurement

#### Outcomes and predictors

Outcome 1: For objective 1, we first examined the determinants of the use of ASHA services. The NFHS-V survey captured the use of ASHA services through multiple questions [24]. (Table 1) The variable representing the use of ASHA services was coded as binary: respondents indicating any utilization of ASHA services during their pre/post-natal period were coded as 1, and 0 otherwise.

**Table 1:** Variable definitions.

Reported use of ASHA services [24]	• If the respondent reported an ASHA assisted her in response to at least one of the following questions: ○ In the last three months, did you meet an ASHA during your (most recent) contact? (No; Yes) ○ Was this pregnancy registered? If yes, was this registered by an ASHA? (No; Yes) ○ During the last three months of this pregnancy, did you meet with ASHA worker? (No; Yes) ○ Who facilitated or motivated you to go to a health facility for delivery? ASHA (No; Yes) ○ Who arranged the transportation to take you to the health facility for delivery? ASHA (No; Yes) ○ Were you ever told by a health worker about any methods of family planning that you can use to avoid pregnancy? If answered yes, then did you get it from? ASHA (No; Yes)
Maternal age category	• Age of the woman at the birth of her last child born. A categorical variable with 5-year age categories
Maternal education	• A categorical variable indicating the highest level of education attended by woman. (No education; Primary; Secondary; Higher)
Place of residence	• A categorical variable indicating whether usual residence is an urban or rural area
6 geographical regions	• A categorical variable indicating 6 regions in India (northern region; north-eastern region; central region; eastern region; western region; southern region)
Wealth Index	• A categorical variable from a scale of 1 to 5 that indicates the wealth of a woman’s household. 1 indicates poorest and 5 indicates richest
Social Status (Caste)	• A categorical variable for what type of caste or tribe a woman belongs to (Higher caste, Schedule Caste, Schedule Tribe, Other Backward Caste)
Religion	• A categorical variable indicating religion followed by woman (Hindu, Muslim, Christian, others)
Birth order (Number of past pregnancies)	• A count variable. Birth order of the last child born in the woman’s life at the time of the survey
Enrollment in a health insurance scheme	• A count variable. Birth order of the last child born in the woman’s life at the time of the survey

Predictor variables for outcome 1: Guided by Anderson’s ‘Behavioural Model of Healthcare Services’ our choice of predictor variables was grounded in three pillars: the health seeker’s predisposition to use services, enabling or impeding factors and the health seeker’s perceived need for care [26]. Consistent with this framework, evidence from India suggests that economic status, caste/ethnicity, education, gender, religion, and culture as the most important structural factors influencing maternal health service use and maternal mortality [15,27]. Further, studies indicate that birth order, distance to the nearest PH centre, perceived quality of care provided at the PH centre, health benefits accrued by mother and children from seeking care, number of ANCs sessions, information about complications during pregnancy, and cultural practices and norms influence the choice of place of delivery [19]. The selection of predictor variables in this analysis thus aligns with Anderson’s theoretical framework, existing evidence, and data availability. Our analysis considered maternal age (grouped into 5-year categories), maternal education, place of residence (urban/rural), wealth index (constructed by the Demographic Health Survey based on household assets, housing materials, water access and sanitation facilities), region of India, religion, caste, birth order and enrollment in a health insurance scheme as the predictor variables. All variables are defined in Table 1.

Outcome 2: In addressing objective 2, we examined the determinants influencing the choice of institution-based delivery versus delivery at home. A new binary variable (0 = No;1 = Yes) for birth in an institution was created based on the place of birth reported by respondents. Instances of deliveries at home (self or parental) were coded as 0, whereas deliveries in a healthcare center, whether public or private, were coded as 1.

Treatment and predictor variable for outcome 2: The use of ASHA services was the primary independent (treatment) variable. Following the same steps to ascertain the choice of predictors, we included registration of pregnancy with CHWs (usually but not necessarily an ASHA), completion of at least one ANC session, and distance from the hospital (as a marker for healthcare access) along with predictors for objective 1, to examine their association with the probability of institution-based deliveries.

### Statistical method

Table 2 provides an overview of study participants categorized by socio-demographic indicators. All results are stratified by reported use of ASHA services, with further segmentation by EAGA states and all India. To answer objective 1, we used a multivariable logistic regression model and examined the association between the use of ASHA services and the underlying socio-demographic characteristics of women respondents. The logistic regression model allowed for the assessment of odds ratios and significance of predictors, which increases ease of interpretation, particularly in view of the research question that aims to examine associations between the dependent (i.e., use of ASHA services) and predictor variables (i.e., the socio-demographic characteristics of women).

**Table 2:** Descriptive characteristics of women by exposure to ASHA workers in India using the NFHS-V survey (2019–21)- Unweighted (By last born child in last 5 years prior to the interview).

*Some key characteristics*	India: N (%)		EAGA: n (%)	
* *	All states	Use of ASHA services Yes: %	EAGA states: %	Use of ASHA services: Yes: %
**Sample size (Ns)**	N = 232,920	n = 147,537 (63.3%)	n = 129,241 (55.5%)	n = 91,203 (70.6%)
**Average age (in years)**	27.3	27.06	26.9	26.9
**Age distribution **				
*15–19*	2.3	2.7	2.3	2.5
*20–24*	28.5	30.1	30.7	31.5
*25–29*	39.7	39.7	40.3	39.9
*30–34*	19.6	18.6	18.1	17.6
*35–39*	7.6	7.0	6.6	6.5
*40–44*	1.8	1.6	1.6	1.6
*45–49*	0.5	0.4	0.4	0.4
**Education**				
*No education*	22.0	22.7	28.9	28.3
*Primary*	12.9	13.4	14.0	14.2
*Secondary*	51.5	52.2	46.2	47.5
*Higher*	13.6	11.7	10.9	10.0
**Place of Residence**				
*Urban*	20.3	15.2	14.0	11.1
*Rural*	79.7	84.8	86.0	88.9
**Wealth Index**				
*Poorest*	27.2	29.5	36.4	37.5
*Poorer*	23.4	24.7	25.7	26.8
*Middle*	19.4	19.2	16.7	16.9
*Richer*	16.8	15.6	12.4	11.7
*Richest*	13.3	11.0	8.8	7.1
**Religion**				
*Hindu*	73.4	76.5	83.8	83.9
*Muslim*	14.4	15.1	14	13.9
*Christian*	8.1	5.0	1.1	1.2
*Others*	4.1	3.5	1.1	1.0
**Caste**				
*Yes i*.*e*., *SC/ST/OBC*	79.0	79.4	82.0	82.4
**Caste (details)**				
*Schedule Caste*	21.7	22.9	23.0	23.5
*Schedule Tribe*	21.4	19.7	15.9	16.8
*Other Backward Caste*	40.3	41.7	46.9	46.6
*Forward caste*	16.6	15.7	14.2	13.1
**Health Insurance**				
*Yes*	73.1	73.0	71.4	72.0
**Birth Order in last 5 years **				
*1*	75.9	80.4	73.7	78.7
*2*	21.6	17.4	23.3	18.7
*3*	2.4	2.1	2.9	2.4
*4*	0.1	0.1	0.1	0.2
*5*	<0.1	<0.1	<0.1	-
*6*	<0.1	<0.1	-	-
**Pregnancy was registered** **with a CHW**				
*Yes*	93.9	96.9	93.4	96.3
**Number of antenatal care visits **				
*No visits*	6.6	4.9	6.6	5.0
*1–3 visits*	35.4	37.1	44.9	45.2
*4 or more visits*	58.0	58.0	48.5	49.8
**Geographical regions **				
*Northern*	16.9	15.9	11.3	8.8
*Central*	27.6	32.6	49.8	52.7
*North-Eastern*	14.7	12.5	8.2	10.0
*Eastern*	19.4	20.6	30.7	28.5
*Western*	8.8	8.1	-	-
*Southern*	12.6	10.3	-	-
**Distance to district hospital **				
*No problem*	36.5	34.3	33.2	31.7
*Not a big problem*	35.5	37.1	37.7	38.9
*Big problem*	28.0	28.6	29.1	29.4

*EAGA- Empowered Action Group States and Assam; ASHA- Accredited Social Health Activists; *SC- Schedule Caste; ST- Schedule Tribe; OBC- Other Backward Caste*.

Second, we estimated the determinants of institution-based delivery, by using propensity score matching (PSM) method to address the selection bias associated with the use of ASHA services [28–30]. The PSM reduces confounding biases in observational studies mimicking the characteristics of a randomized controlled trial [28]. This method enabled us to address the observable selection bias in our study population [31,32]. For this study, participants were matched based on maternal age category, education status, wealth index, religion, caste, place of residence, birth index of last-born child, EAGA status and region in India. Propensity scores were then derived using a greedy, one-to-one match with 0.2 caliper distance (i.e., the standard deviation of the logit of the propensity score, to estimate the average treatment effect on the treated) [28–30]. To ensure that the propensity score’s distribution was similar for treatment and control groups, we assessed the quality of matched pairs by evaluating standardized mean differences between predictors to achieve balance before and after matching [28]. A standardized mean difference threshold of less than 10% was considered an acceptable balance [33]. Sensitivity analyses were conducted to enhance the robustness of the findings. Three iterations of matching based on socio-demographic characteristics were performed to examine the overlap between the matched cohorts. (S1 Appendix) Multiple iterations of PSM were performed, and different matching algorithms, such as nearest neighbour with replacement, radius matching, and kernel matching, were employed to assess the consistency of results [33]. The results of these sensitivity analyses, including balance checks, were documented, and presented in supplementary materials for transparency and completeness. (Refer S2 and S3 Tables and S1–S3 Figs) Additionally, to examine the validity of the results, a similar PSM model was performed using a previous survey round of the NFHS (NFHS-IV (2015–16)): findings were similar to NFHS-V (Results available upon request).

Finally, data were restricted to 154,544 matched observations to run a generalized estimating equation (GEE) model to assess the predictors of institution-based deliveries. All analyses were performed in STATA 17 SE (StataCorp).

## Results

The study comprised 232,920 women aged 15–49 years who reported the birth of their last child within the five years preceding the interview (Table 2). Of these, more than half (55.5%) lived in the nine EAGA states. Nationally, 63.3% reported using ASHA services during their most recent pregnancy, with a higher prevalence observed within the EAGA states (70.6%). A substantial subset of the study population (All India = 70.5%; EAGA = 73.3%) were women under 30 years of age. A minority, accounting for 13.6% of all India and 11.9% in EAGA states, had attained post-secondary or other higher education. Most participants (All India = 79.7%; EAGA = 86.0%), reported residing in rural areas. Our sample comprised 73.4% Hindus (EAGA = 83.4%), and the majority (All India = 79.0%; EAGA = 82.0%) reported belonging to low socioeconomic status groups such as Schedule Caste, Schedule Tribe or Other Backward Caste. Half (50.6%) of the respondents were within the poor or poorest income quintile in India, with a higher proportion (62.1%) observed in EAGA states. Health insurance enrollment was reported by 73.1% all India (EAGA = 71.4%), while 93.9% all India (EAGA = 93.4%) had registered their last pregnancy. Only 58.0% of women reported completion of the recommended four or more ANC visits [34], and even fewer in EAGA states (48.5%). Additionally, while the overall prevalence of institution-based delivery in India is 88.6% (EAGA = 84.0%), we observe large variations in the prevalence of institution-based deliveries by socioeconomic status and in EAGA states (Fig 1).

The results from the multivariate logistic regression suggest that in all India context, women with primary and secondary education were at least 1.1 times more likely to use ASHA services, compared to those with no education. However, women with the highest education (post-secondary or higher education level) were less likely to use ASHA services (Table 3). Age emerged as a significant predictor for the use of ASHA services with women in the relatively younger age group of 15–19 years, having higher odds (OR = 1.94; 95% CI = 1.38–1.94) of using ASHA services compared to women aged 45–49 years. Additionally, when compared to the poorest wealth quintile, women in higher income quintiles (fourth: OR = 0.80; 95% CI = 0.77–0.82 or fifth: OR = 0.71; 95% CI = 0.69–0.74) were less likely to use ASHA services. Moreover, Muslim women were more likely (OR = 1.26; 95% CI = 1.23–1.30), while Christian women were less likely (OR = 0.68; 95% CI = 0.64–0.73) to use ASHA services in comparison to Hindus. Other factors influencing the use of ASHA services included rural residence (OR = 2.14; 95% CI = 2.09–2.19) belonging to low socioeconomic status groups such as Scheduled Caste (OR = 1.20; 95% CI = 1.17–1.24), Scheduled Tribe (OR = 1.27; 95% CI = 1.22–1.32) or Other Backward Caste (OR = 1.07, 95% CI = 1.04–1.10). Finally, women with any health insurance (OR = 0.96; CI = 0.94–0.98) and those with one or more previous births were significantly less likely to use ASHA services (OR = 0.45; CI = 0.44–0.46). The results from EAGA states followed similar associations as found for all of India, with only marginal variations (Table 3).

**Table 3:** Analysis of the association between exposure to ASHA services for the most recent birth and individual characteristics using logistic regressions using NFHS-V (2019–21).

Use of ASHA services	India (n = 220,631)			EAGA (n = 123,493)		
	Odds Ratio	95% CI	*p-value*	Odds Ratio	95% CI	*p-value*
**Education (Ref: No Education)**						
*Primary*	1.13	(1.10–1.17)	<0.001	1.14	(1.09–1.18)	<0.001
*Secondary*	1.15	(1.12–1.18)	<0.001	1.20	(1.16–1.24)	<0.001
*Higher*	0.90	(0.87–0.93)	<0.001	0.98	(0.93–1.03)	0.382
**Age category (Ref: Ages 45–49 years)**						
*15–19*	1.94	(1.38–1.94)	<0.001	1.27	(1.03–1.57)	0.024
*20–24*	1.71	(1.24–1.71)	<0.001	1.35	(1.12–1.64)	0.002
*25–29*	1.62	(1.18–1.62)	<0.001	1.34	(1.11–1.62)	0.003
*30–34*	1.44	(1.05–1.44)	0.011	1.22	(1.01–1.48)	0.042
*35–39*	1.41	(1.02–1.41)	0.026	1.23	(1.01–1.49)	0.040
*40–44*	1.40	(0.99–1.4)	0.068	1.15	(0.93–1.42)	0.209
**Wealth Index (Ref: Poorest)**						
*Poor*	0.96	(0.93–0.99)	0.005	1.05	(1.01–1.09)	0.006
*Middle*	0.85	(0.83–0.88)	<0.001	1.01	(0.97–1.05)	0.550
*Rich*	0.80	(0.77–0.82)	<0.001	0.89	(0.85–0.93)	<0.001
*Richest*	0.71	(0.69–0.74)	<0.001	0.69	(0.66–0.73)	<0.001
**Place of Residence (Ref: Urban)**						
*Rural*	2.14	(2.09–2.19)	<0.001	1.98	(1.91–2.05)	<0.001
**Religion (Ref: Hindu)**						
*Muslim*	1.26	(1.23–1.30)	<0.001	0.97	(0.93–1.01)	0.092
*Christian*	0.68	(0.64–0.73)	<0.001	1.15	(0.97–1.38)	0.11
*Others*	1.04	(0.98–1.10)	0.245	0.81	(0.71–0.93)	0.003
**Caste (Ref: Forward caste)**						
*Schedule Caste*	1.20	(1.17–1.24)	<0.001	1.30	(1.24–1.35)	<0.001
*Schedule Tribe*	1.27	(1.22–1.32)	<0.001	1.39	(1.31–1.46)	<0.001
*Other Backward Caste*	1.07	(1.04–1.10)	<0.001	1.19	(1.15–1.24)	<0.001
**Health insurance (Ref: No)**						
*Yes*	0.96	(0.94–0.98)	<0.001	0.81	(0.79–0.84)	<0.001
**Birth order of child (Ref: First child)**						
*2*	0.45	(0.44–0.46)	<0.001	0.41	(0.40–0.43)	<0.001
*3*	0.50	(0.47–0.53)	<0.001	0.46	(0.43–0.49)	<0.001
*4*	0.50	(0.39–0.64)	<0.001	0.50	(0.38–0.66)	<0.001
*5*	2.59	(0.23–29.78)	0.444	1.26	(0.09–17.51)	0.861
**EAGA state (Ref: No)**						
*Yes*	1.67	(1.64–1.71)	<0.001	-	-	-
**Constant**	1.00	(0.93–1.07)	0.918	1.33	(1.20–1.48)	<0.001

Before applying PSM, the proportion of women who reported having an institution-based delivery was significantly higher (88.6%) among women who used ASHA services than those who did not (83.2%) (Table 4). Matching procedures increased the positive effect of the use of ASHA services on institution-based delivery (Table 4). This suggests that the observed differences in the unmatched sample were, in part, due to the variation in the distribution of socioeconomic attributes between women using ASHA services and those who did not. The average treatment effect on treated (ATT) due to the use of ASHA services on institution-based deliveries was found to be 5.1% in all India and 7.4% in EAGA states after matching for observable characteristics. The ATT is a measure of the difference in outcome in the treatment group to a similar unit in the control group.

**Table 4:** The average treatment effect on the treated for use of ASHA services on institution-based delivery.

	Means before matching (%)				Average treatment effect			
Institution-based delivery	Used ASHA services	Not used ASHA services	Difference	*p-value*	ATT	95% CI	SE	*p-value*
All India	88.0	83.2	4.7	<0.001	5.1	4.8–5.5	0.17909	<0.001
EAGA states	86.4	80.6	5.8	<0.001	7.4	6.9–7.9	0.27168	<0.001

Using PSM and restricting the sample to matched observations only, the GEE model examined the factors associated with the likelihood of institution-based deliveries. The use of ASHA services increased the odds of institution-based delivery by 1.58 (95%CI = 1.51–1.65) for all India and by 1.78 (95%CI = 1.68–1.89) in EAGA states (Table 5). Younger women with higher education and wealth index were more likely to have an institution-based delivery. Urban/rural residence was not a statistically significant predictor of institution-based delivery either across India or in EAGA states. Belonging to a religious minority (Muslims, Christians, and others) or identifying as a low socioeconomic status group substantially reduced the odds of having an institution-based delivery compared to reference populations. Having health insurance (all India: OR = 1.4; 95% CI = 1.3–1.5; EAGA states: OR = 1.7; 95%CI = 1.6–1.8) significantly improved the odds of having an institution-based birth. Similarly, women who registered their pregnancy and attended at least one ANC session were twice as likely to have an institution-based birth compared to those not, both for all India and in EAGA states. The ease of reaching the nearest health facility, a marker of access, was a significant predictor of an institution-based delivery. Compared to those who indicated facing a ‘big problem to reach nearest health facility’, those who indicated having ‘no problem to reach’ had higher odds of institution-based birth by almost 1.4 times for all India and EAGA states. Unfortunately, the role of conditional cash transfers like JSY in motivating women to have an institution-based birth could not be examined, as the transfers were limited to women who had an institution-based delivery.

**Table 5:** Association between institutional delivery and exposure to ASHAs adjusting for socio-demographic characteristics using multivariate logistic regression (After matching).

Institution-based delivery	India (n = 112,294; Pairs: 68,217)			EAGA (n = 47,770, Pairs:29,710)		
	Odds Ratio	95% CI	*p-value*	Odds Ratio	95% CI	*p-value*
**Use of ASHA services (Ref: No) **						
Yes	1.58	(1.51–1.65)	<0.001	1.78	(1.68–1.89)	<0.001
**Education (Ref: No Education) **						
*Primary*	1.23	(1.16–1.31)	<0.001	1.29	(1.19–1.39)	<0.001
*Secondary*	2.04	(1.94–2.15)	<0.001	1.75	(1.63–1.88)	<0.001
*Higher*	4.31	(3.87–4.81)	<0.001	3.74	(3.23–4.32)	<0.001
**Age category (Ref: Ages 45–49 years) **						
*15–19*	1.75	(1.41–2.17)	<0.001	1.64	(1.21–2.21)	0.001
*20–24*	1.61	(1.34–1.93)	<0.001	1.63	(1.27–2.08)	<0.001
*25–29*	1.33	(1.12–1.59)	0.002	1.29	(1.01–1.64)	0.040
*30–34*	1.34	(1.12–1.61)	0.001	1.25	(0.98–1.59)	0.076
*35–39*	1.25	(1.04–1.5)	0.012	1.11	(0.87–1.43)	0.394
*40–44*	1.05	(0.86–1.29)	0.599	0.93	(0.71–1.23)	0.622
**Wealth Index (Ref: Poorest) **						
*Poor*	1.66	(1.58–1.75)	<0.001	1.6	(1.49–1.72)	<0.001
*Middle*	2.45	(2.29–2.61)	<0.001	2.04	(1.86–2.23)	<0.001
*Rich*	3.61	(3.32–3.92)	<0.001	2.59	(2.32–2.88)	<0.001
*Richest*	4.81	(4.29–5.38)	<0.001	3.81	(3.28–4.43)	<0.001
**Place of Residence (Ref: Urban) **						
*Rural*	0.97	(0.91–1.02)	0.11	0.96	(0.88–1.04)	0.336
**Religion (Ref: Hindu) **						
*Muslim*	0.64	(0.59–0.68)	<0.001	0.61	(0.57–0.66)	<0.001
*Christian*	0.31	(0.29–0.33)	<0.001	0.55	(0.45–0.67)	<0.001
*Others*	0.72	(0.65–0.79)	0.001	0.76	(0.62–0.92)	0.006
**Caste (Ref: Forward caste) **						
*Schedule Caste*	1.04	(0.96–1.12)	0.348	0.86	(0.78–0.95)	0.002
*Schedule Tribe*	0.77	(0.71–0.83)	<0.001	0.74	(0.67–0.82)	<0.001
*Other Backward Caste*	1.21	(1.13–1.29)	<0.001	1.02	(0.94–1.1)	0.681
**Health insurance (Ref: No) **						
*Yes*	1.39	(1.33–1.46)	<0.001	1.65	(1.55–1.76)	<0.001
**Birth order of child (Ref: First child) **						
*2*	0.87	(0.79–0.95)	<0.001	0.8	(0.73–0.89)	<0.001
*3*	0.52	(0.22–1.22)	0.065	0.59	(0.24–1.45)	0.252
**EAGA state (Ref: No) **						
*Yes*	0.60	(0.57–0.63)	<0.001	-	-	-
**Registered pregnancy (Ref: No) **						
*Yes*	2.10	(1.98–2.24)	<0.001	1.7	(1.57–1.85)	<0.001
**At least 1 antenatal care session (Ref: No) **						
*Yes*	2.48	(2.33–2.64)	<0.001	2.23	(2.05–2.43)	<0.001
**Reaching nearest healthcare facility (Ref: Big problem) **						
*No problem*	1.38	(1.31–1.45)	<0.001	1.35	(1.26–1.45)	<0.001
*Not a big problem*	1.17	(1.12–1.23)	<0.001	1.21	(1.13–1.3)	<0.001
**Constant**	0.44	(0.35–0.54)	<0.001	0.41	(0.31–0.54)	<0.001

The sensitivity analysis underscored the significance of matching socio-demographic characteristics in addressing the issue of self-selection. Matching effectively reduced the overall standardized mean differences between women who used ASHA services and those who did not, bringing them to an acceptable threshold for the final model. (S4 and S5 Tables)

## Discussion

Prior investigations in this field were limited by geographic scope, with only a few utilizing national survey data for India or adequately addressing selection bias in the analysis. Our study examined the association between the use of ASHA services and the uptake of institution-based deliveries in India. We focussed on low-performing EAGA states while addressing the inherent selection bias in the population using the latest round of the NFHS.

This study contributes compelling evidence that supports the instrumental role of ASHAs in mitigating existing disparities in primary healthcare access and improving health equity in the uptake of institution-based deliveries in India [15,16,35]. We observed a 1.7-fold higher probability for the use of ASHA services in EAGA states compared to non-EAGA states, with a two-fold increased likelihood in rural compared to urban areas. In a recent study, Ali and colleagues (2020) highlighted significant disparities in per capita accessibility of primary care centers and the availability of adequately trained medical personnel in rural areas and EAGA states [36]. Our study findings lend additional support to the heightened reliance on non-traditional healthcare providers, such as ASHAs, among women seeking essential primary healthcare services. These observations emphasize the critical role of ASHAs in bridging healthcare gaps and meeting the unique needs of communities facing challenges in accessing traditional healthcare resources. By doing so, ASHAs contribute significantly to reducing healthcare inequalities and promoting inclusive healthcare practices.

Additionally, we find that women from religious minority groups and low socioeconomic status groups are more likely to use ASHA services, a pattern that is heightened among younger women belonging to lower wealth indices [15,16,37]. Furthermore, compared to their forward caste counterparts, the preference for ASHA services among women identifying as Schedule Caste, Schedule Tribe and Other Backward Caste underscores the program’s efficacy in reaching and catering to traditionally underserved and marginalized populations and improving inequities in accessing essential healthcare services [15]. Increased penetration of government programs like health insurance schemes and the introduction of safe motherhood schemes like the JSY have notably improved facility-based deliveries, in India [15,19]. We find that the use of ASHA services increased the likelihood of an institution-based delivery by at least 50% throughout India and about 80% in EAGA states, even after addressing the selection bias. Serving as front-line health workers, ASHAs facilitate a continuum of care for underserved marginalized populations with high mortality burden. They also bridge the outreach gap between primary health centres and health-seeking communities particularly for expectant mothers in rural communities, improving overall maternal and child outcomes [1]. These study findings resonate with the broader conceptualization of CHW programs in LMICs, which are intentionally designed to address healthcare needs and reduce health inequities within populations traditionally marginalized by existing health systems [10]. Our study empirically substantiates that the ASHA program has successfully reached its target population and is consistent with past evidence presented by Agarwal (2019) [15].

Furthermore, our study highlights that while the relative utilization of ASHA services is greater for underserved groups belonging to low socioeconomic status groups, the use of ASHA services does not fully eliminate disparities in the uptake of institution-based deliveries. Past studies by Sarkar (2018) and Ali (2020) have identified illiteracy and poor economic status to be major contributors to creating inequality in achieving national targets for health indicators such as institution-based deliveries [36,38]. Thus, despite the presence of ASHAs, the socioeconomic status of women continues to be one of the biggest drivers of institution-based deliveries [15,19,38].

Recent evaluations of the ASHA program highlight operational challenges, including insufficient contact with healthcare-seeking women, high workload, inadequate training, supervision gaps, and low, uneven compensation [39,40]. ASHAs operate under a performance-based incentive program, linking monetary rewards to measurable tasks. Given their crucial role, it is essential to provide them with fair pay and conducive work environments to maintain the quality-of-service provision by ASHAs and for overall health system robustness and resilience [39,40]. To achieve Universal Health Care where “no one is left behind”, the role of ASHAs in India is indispensable [41]. Their contribution to improving maternal outcomes is being acknowledged, globally, with ASHAs recently being awarded the Global Health Leaders Award by the World Health Organization [42]. However, these frontline workers, predominantly female workforce need to be acknowledged, adequately and fairly integrated into health systems with appropriate compensation.

Finally, our study sheds light on the impact of ASHA services, enrollment into health insurance schemes, and registration of pregnancy, on increasing institution-based deliveries and potentially improving overall maternal care service utilization. However, we find that this improvement does not completely eliminate inequities, due to additional barriers faced by marginalized women that influence their choice for institution-based deliveries. Cultural and structural barriers, such as attitudes of household members towards health-seeking practices, traditional belief systems, higher out-of-pocket expenditures, mode of transportation, penetration and access to healthcare facilities, and distance to the nearest hospitals can all influence the uptake of institution-based deliveries [6,35]. Policy and program development that aim to enhance maternal and child health outcomes need to address these barriers to access and utilization of essential care to ensure more equitable access to health care.

This study has some limitations. We assume that respondents who use ASHA services receive a continuum of care during their pregnancy and confer benefits that increase the likelihood of institutional birth. However, the use of healthcare during this period may be inconsistent. The cross-sectional nature of NFHS data makes it challenging to confirm a continuum of care, which is a common challenge in similar studies [15,43]. The global COVID-19 pandemic, starting in March 2020, disrupted NFHS-V data-collection activities in seven out of nine EAGA states in India. Notably, data from non-EAGA states, as well as Bihar and Assam were collected before the pandemic (June 2019 to January 2020). For the other seven EAGA states, data collection occurred in two phases: a) January 2020 to March 2020 (pre-COVID-19 lockdowns) and b) between December 2020 and March 2021 (post-COVID-19 lockdown). Despite a small impact on our sample population, with 5.6% reporting deliveries during the lockdown, some effects of this exogenous shock may be present. We were unable to incorporate the impact of JSY within our model, and thus cannot separate the effect of the use of ASHA services and the impact of the JSY scheme on the likelihood of institution-based deliveries. Unobserved variables such as family members’ attitudes (e.g., mother-in-law) towards health seeking, could influence both the use of ASHA services and uptake of institution-based deliveries [19]. Finally, our use of PSM to address selection bias, while allowing a quasi-experimental design, resulted in the exclusion of unmatched observations, potentially impacting results as these excluded observations may differ in characteristics from those that were included.

## Conclusion

In the face of recent global exogenous shocks, notably the COVID-19 pandemic, women’s ability to seek health care has been significantly impacted. It has also further exacerbated resource constraints within the health systems in India and other LMICs. The pivotal role of ASHAs as a bridge between primary healthcare providers and healthcare seekers is becoming increasingly evident.

Our study reinforces the effectiveness of ASHAs in increasing the uptake of maternal services, particularly institution-based deliveries. This underscores the necessity for continuous, systematic investments in the ASHA program. Investments in the development of targeted strategies such as comprehensive training programs for ASHAs, robust supervision mechanisms, and ensuring fair compensation, could substantially improve the program’s impact. Strengthening the ASHA program is vital for advancing child and maternal health outcomes, particularly in settings reliant on the community health worker model. In an ever-changing global health context, a sustained commitment to empowering ASHAs remains integral to ensuring resilient and effective maternal and child healthcare.

## Supporting information

S1 Fig Overlap between women who used ASHA services and not, using States from Iteration 1 before and after matching. (DOCX)

S2 Fig Overlap between women who used ASHA services and not, using EAGA states and Regions from Iteration 2 before and after matching. (DOCX)

S3 Fig Overlap between women who used ASHA services and not, using EAGA states and Regions from Iteration 3 before and after matching. (DOCX)

S1 Table STROBE statement—Checklist of items that should be included in reports of cross-sectional studies. (DOCX)

S2 Table Socio-economic indicators matched on in Propensity Score Matching. (DOCX)

S3 Table Results from 3 different propensity score matching models. (DOCX)

S4 Table The balance between predictor variables for institution-based deliveries by exposure to ASHAs before and after propensity score matching (All India). (DOCX)

S5 Table The balance between predictor variables for institution-based deliveries by exposure to ASHAs before and after propensity score matching (EAGA states). (DOCX)

S1 Appendix Iterations of propensity score matching model. (DOCX)
