# Supplementary material for: Association between the use of Accredited Social Health Activist (ASHA) services and uptake of institutional deliveries in India
Source: PLOS Glob Public Health. 2024 Jan 16;4(1):e0002651. doi: 10.1371/journal.pgph.0002651 (PMC10790990; doi:10.1371/journal.pgph.0002651)
Supplement: S3 Fig — (DOCX) [file pgph.0002651.s003.docx]

**S3 Fig: Overlap between women who used ASHA services and not, using EAGA states and Regions from Iteration 3 before and after matching**
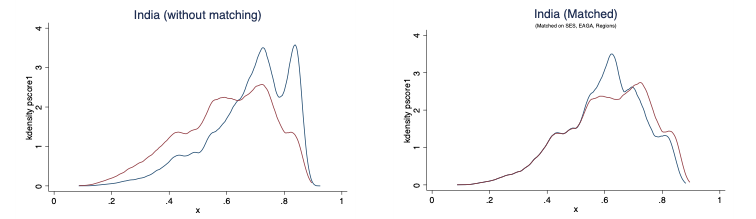


**
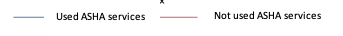
**
