# Supplementary material for: Association between the use of Accredited Social Health Activist (ASHA) services and uptake of institutional deliveries in India
Source: PLOS Glob Public Health. 2024 Jan 16;4(1):e0002651. doi: 10.1371/journal.pgph.0002651 (PMC10790990; doi:10.1371/journal.pgph.0002651)
Supplement: S2 Table — (DOCX) [file pgph.0002651.s005.docx]

**S2 Table: Socio-economic indicators matched on in Propensity Score Matching**

| **socio-demographic characteristics matched on** | **Iteration 1** | **Iteration 2** | **Iteration 3 (Chosen model)** |
| --- | --- | --- | --- |
| Respondent’s age | ✓ | ✓ | ✓ |
| Respondent’s Education | ✓ | ✓ | ✓ |
| Respondent’s wealth index | ✓ | ✓ | ✓ |
| Residence (Rural/Urban) | ✓ | ✓ | ✓ |
| Religion | ✓ | ✓ | ✓ |
| Caste | ✓ | ✓ | ✓ |
| Health insurance status | ✓ | ✓ | ✓ |
| Birth index of last-born child | ✓ | ✓ | ✓ |
| Registered pregnancy | ✓ | ✓ | X |
| At least one antenatal care | ✓ | ✓ | X |
| Received institutional advice | ✓ | ✓ | X |
| Geographic indicator (s) | States | EAGA states & Regions | EAGA states & Regions |
