# Supplementary material for: Association between the use of Accredited Social Health Activist (ASHA) services and uptake of institutional deliveries in India
Source: PLOS Glob Public Health. 2024 Jan 16;4(1):e0002651. doi: 10.1371/journal.pgph.0002651 (PMC10790990; doi:10.1371/journal.pgph.0002651)
Supplement: S3 Table — (DOCX) [file pgph.0002651.s006.docx]

**S3 Table: Results from 3 different propensity score matching models**

|  | Iteration 1 | | | Iteration 2 | | | Iteration 3 | | |
| --- | --- | --- | --- | --- | --- | --- | --- | --- | --- |
| No. of Observations | Used ASHA services | |  | Used ASHA services | |  | Used ASHA services | |  |
|  | No | Yes | Total | No | Yes | Total | No | Yes | Total |
| Matched | 20,800 | 82954 | 103754 | 34,163 | 96317 | 130480 | 8,111 | 70265 | 78376 |
| Unmatched | 64,583 | 64583 | 129166 | 51,220 | 51220 | 102440 | 77,272 | 77272 | 154544 |
|  | 85,383 | 147,537 | 232,920 | 85,383 | 147,537 | 232,920 | 85,383 | 147,537 | 232,920 |
| % observations dropped due to matching | | 44.50% | | 56.10% | | | 33.60% | | |
