# Supplementary material for: Association between the use of Accredited Social Health Activist (ASHA) services and uptake of institutional deliveries in India
Source: PLOS Glob Public Health. 2024 Jan 16;4(1):e0002651. doi: 10.1371/journal.pgph.0002651 (PMC10790990; doi:10.1371/journal.pgph.0002651)
Supplement: S4 Table — (DOCX) [file pgph.0002651.s007.docx]

**S4 Table: The balance between predictor variables for institution-based deliveries by exposure to ASHAs before and after propensity score matching (All India)**

| *Matched characteristics* | **India: N (%)** | | | | | | |  |
| --- | --- | --- | --- | --- | --- | --- | --- | --- |
|  | **Unmatched** | | | | **Matched** | | | |
|  | **All states** | **Use of ASHA services** | | | **All states** | **Use of ASHA services** | | |
|  | *N= 232,920 (100)* | *No:  n= 85,383 (36.7)* | *Yes:  n= 147,537 (63.3)* | *Std Diff** | *N= 154,544 (66.4)* | *No:  n= 77,272 (50)* | *Yes:  n= 77,272 (50)* | *Std Diff* |
| **Average age (years)** | 27.3 | 27.8 | 27.1 | 0.1 | 27.7 | 27.7 | 27.7 | <0.1 |
| **Education (%)** |  |  |  |  |  |  |  |  |
| *No education* | 22.0 | 34.3 | 65.7 | 0.1 | 21.1 | 50.6 | 49.4 | -<0.1 |
| *Primary* | 12.9 | 34.5 | 65.5 |  | 12.6 | 49.5 | 50.5 |  |
| *Secondary* | 51.5 | 35.8 | 64.2 |  | 50.6 | 50.4 | 49.6 |  |
| *Higher* | 13.6 | 45.6 | 54.4 |  | 15.8 | 48.5 | 51.5 |  |
| **Rural residence (%)** | 80.0 | 70.9 | 84.9 | -0.3 | 74.5 | 74.7 | 74.5 | <0.1 |
| **Wealth Index (%)** |  |  |  |  |  |  |  |  |
| *Poorest* | 27.2 | 31.5 | 68.5 | 0.2 | 23.3 | 52.7 | 47.3 | -<0.1 |
| *Poorer* | 23.4 | 33.1 | 66.9 |  | 21.8 | 50.5 | 49.5 |  |
| *Middle* | 19.4 | 37.0 | 63.0 |  | 20.1 | 49.4 | 50.6 |  |
| *Richer* | 16.8 | 40.9 | 59.2 |  | 19.1 | 47.9 | 52.1 |  |
| *Richest* | 13.3 | 47.7 | 52.3 |  | 15.7 | 48.6 | 51.4 |  |
| **Religion (%)** |  |  |  |  |  |  |  |  |
| *Hindu* | 73.4 | 34.0 | 66.0 | 0.2 | 72.8 | 47.4 | 52.6 | 0.2 |
| *Muslim* | 14.4 | 33.7 | 66.3 |  | 12.5 | 47.0 | 53.0 |  |
| *Christian* | 8.1 | 60.9 | 39.1 |  | 10.0 | 70.1 | 29.9 |  |
| *Others* | 4.1 | 46.3 | 53.7 |  | 4.7 | 55.9 | 44.1 |  |
| **ST/SC/OBC** (%)** | 79.0 | 78.4 | 79.4 | -<0.1 | 81.6 | 82.9 | 80.3 | <0.1 |
| **Have health insurance (%)** | 26.9 | 26.8 | 27.0 | -<0.1 | 27.9 | 27.4 | 28.4 | -<0.1 |
| **Average children every born (Nos.)** | 1.3 | 1.4 | 1.2 | 0.3 | 1.4 | 1.4 | 1.4 | -<0.1 |
| *Std Diff- Standard Difference; **ST/SC/OBC- Respondents identified as Schedule Tribe, Schedule caste or Other Backward Caste; †JSY: Janani Suraksha Yojna; § ANC: Ante-natal Care | | | | | | | | |
