# Supplementary material for: Association between the use of Accredited Social Health Activist (ASHA) services and uptake of institutional deliveries in India
Source: PLOS Glob Public Health. 2024 Jan 16;4(1):e0002651. doi: 10.1371/journal.pgph.0002651 (PMC10790990; doi:10.1371/journal.pgph.0002651)
Supplement: S5 Table — (DOCX) [file pgph.0002651.s008.docx]

**S5 Table: The balance between predictor variables for institution-based deliveries by exposure to ASHAs before and after propensity score matching (EAGA states)**

| *Matched characteristics* | **EAGA states: N (%)** | | | | | | |  |
| --- | --- | --- | --- | --- | --- | --- | --- | --- |
|  | **Unmatched** | | | | **Matched** | | | |
|  | **All states** | **Use of ASHA services** | | | **All states** | **Use of ASHA services** | | |
|  | *n= 129,241 (55.5)* | *No:  n= 38,038 (29.4)* | *Yes:  n= 91,203 (70.6)* | *Std Diff** | *n= 71,688 (55.5)* | *No:  n= 35,844 (50)* | *Yes:  n= 35,844 (50)* | *Std Diff* |
| **Average age (years)** | 26.9 | 27.2 | 26.9 | <0.1 | 27.3 | 27.2 | 27.6 | -<0.1 |
| **Education (%)** |  |  |  |  |  |  |  |  |
| *No education* | 28.9 | 30.9 | 69.1 | <0.1 | 30.3 | 50.6 | 49.4 | -<0.1 |
| *Primary* | 14.0 | 28.1 | 71.9 |  | 14.0 | 47.7 | 52.3 |  |
| *Secondary* | 46.2 | 27.4 | 72.6 |  | 41.8 | 51.6 | 48.4 |  |
| *Higher* | 10.9 | 35.8 | 64.2 |  | 13.9 | 46.3 | 53.7 |  |
| **Rural residence (%)** | 86.0 | 78.9 | 88.9 | -0.3 | 78.8 | 80.3 | 77.2 | <0.1 |
| **Wealth Index (%)** |  |  |  |  |  |  |  |  |
| *Poorest* | 36.4 | 27.2 | 72.8 | 0.2 | 31.0 | 54.8 | 45.3 | -0.1 |
| *Poorer* | 25.7 | 26.4 | 73.6 |  | 23.5 | 49.7 | 50.3 |  |
| *Middle* | 16.8 | 28.9 | 71.1 |  | 17.2 | 48.7 | 51.3 |  |
| *Richer* | 12.4 | 33.4 | 66.6 |  | 15.5 | 45.3 | 54.7 |  |
| *Richest* | 8.8 | 43.0 | 57.0 |  | 12.9 | 46.6 | 53.4 |  |
| **Religion (%)** |  |  |  |  |  |  |  |  |
| *Hindu* | 83.8 | 29.4 | 70.7 | <0.1 | 82.6 | 51.4 | 48.6 | -<0.1 |
| *Muslim* | 14.0 | 30.0 | 70.0 |  | 15.3 | 42.4 | 57.6 |  |
| *Christian* | 1.1 | 22.6 | 77.4 |  | 1.0 | 44.1 | 55.9 |  |
| *Others* | 1.1 | 35.1 | 64.9 |  | 1.2 | 51.1 | 48.9 |  |
| **ST/SC/OBC** (%)** | 82.0 | 81.0 | 82.5 | -<0.1 | 80.1 | 84.1 | 76.2 | 0.2 |
| **Have health insurance (%)** | 28.6 | 30.0 | 28.0 | -<0.1 | 30.7 | 29.4 | 32.0 | -<0.1 |
| **Average children every born (Nos.)** | 1.3 | 1.4 | 1.2 | 0.3 | 1.4 | 1.4 | 1.5 | -<0.1 |
| *Std Diff- Standard Difference; **ST/SC/OBC- Respondents identified as Schedule Tribe, Schedule caste or Other Backward Caste; †JSY: Janani Suraksha Yojna; § ANC: Ante-natal Care | | | | | | | | |
