# Supplementary material for: Association between the use of Accredited Social Health Activist (ASHA) services and uptake of institutional deliveries in India
Source: PLOS Glob Public Health. 2024 Jan 16;4(1):e0002651. doi: 10.1371/journal.pgph.0002651 (PMC10790990; doi:10.1371/journal.pgph.0002651)
Supplement: S1 Appendix — (DOCX) [file pgph.0002651.s009.docx]

**S1 Appendix: Iterations of propensity score matching model**:

We tried three different iterations using greedy, one-to-one nearest neighbour matching with a caliper distance of 0.2 logit of propensity scores to match women respondents who used ASHA services compared to women who reported not.

- S2 Table presents the details of the matching characteristics of the three iterations.
- S3 Table presents the number of cases matched by the three iterations. Matching using iterations 1 and 2 dropped 44.5% and 56.1% of the total sample of observations.
- S1 Fig – S3 Fig presents the overlap of the two cohorts (i.e., used ASHA services and not used ASHA services) after matching the socio-demographic characteristics.

Sensitivity analyses

- The balance was also checked for matching algorithms, including nearest neighbour with replacement, radius matching, and Kernel matching.
- We also conducted a similar analysis using a previous round of the National Family Health Survey (i.e., National Family Health Survey- IV (2015-16)) and found that the results align with our study. Results can be presented on request.
